# Supplementary material for: Microvascular Complications and Cancer Risk in Type 2 Diabetes: A Population-Based Study
Source: Cancers (Basel). 2025 May 23;17(11):1760. doi: 10.3390/cancers17111760 (PMC12153810; doi:10.3390/cancers17111760)
Supplement: Supplementary file 1 [file cancers-17-01760-s001.zip › cancers-3642671-supplementary.pdf]

Additional file S1.

Table S1 Diseases and related ICD-9-CM, ICD-10-CM codes

Disease	ICD-9-CM codes	ICD-10-CM codes
Type 2 diabetes	250.xx, except 250.1x	E11
Type 1 diabetes	250.1x	E10
Chronic kidney disease	250.4, 403-404, 585-586, 581.8, 593.9, 791.0, V42.0, V45.1, V56.0, V56.8, ICD-9-PCS: 39.27, 39.42-39.43, 39.49-39.50, 39.53, 39.93-39.95	I12, I13, N08, N18, N19, N29, E10.2, E11.2, E13.2, N02.8, N04.7, N04.8, N18.9, N28.9, R80.8, R80.9, N18.1-N18.3, R80.0-R80.3, Z94.0, Z99.2, Z94.0, E10.65, E11.65, Z49.31, Z49.32
Diabetic retinopathy	362.01、362.02、362.07, 362.53、362.83、364.42、379.23、369, ICD-9-PCS:14.2-14.5、14.7	E11.31-E11.34, E11.35, H54, H43.1, H35.35, H35.81, H21.1X, ICD-10-PCS: 085F3ZZ, 08H031Z, 08H0X1Z, 08H131Z, 08H1X1Z
Diabetic neuropathy	250.6, 302.72, 337.0, 337.1, 354-355, 356.9, 357.2, 358.1, 458, 536.3, 564.5, 595.54, 607.84, 713.5, 729.2, 951.0, 951.1, 951.3	E08-11, E13.49, F52.21, F52.22, F52.8, G56-59, G60.9, G70.8, G70.81, G73.1, G73.3, G90.01, G90.09, G99.0, I95, K59.1, K31.84, M14, M54.10, M54.18, M79.2, N31.0, N31.1, N31.9, N52, S04
Oral cavity cancer	140-149	C00-C14
Thyroid cancer	193	C73
Breast cancer	174-175	C50
Respiratory organ cancer	160-165	C30-C39
Digestive organ cancer	150-159	C15-C26
Lymphoid tissues cancer	200-208	C81-C96
Female genital organ cancer	179-184	C51-C58
Male genital organ cancer	185-187	C60-C63
Cancer related death	140-209,230-239	C00-D2, D3B-D9, D37-D49
Overweight	278.02, 783.1, V85.2	R63.5
Obesity	278.02, 783.1, V85.2, 278.00, 649.1, V77.8, V85.3, 278.01, 649.2, V45.86, V85.4	R63.5, E66.09, E66.1, E66.8, E66.9, Z13.89, E66.01, E66.2
Smoking status	305.1, 649.0, V15.82	F17.200, F17.201, F17.210, F17.220, F17.221, F17.290, F17.291, Z87.891
Hypertension	401–405, A26	I10, I11, I12, I13, I15, N26

Dyslipidemia	272	E71.30, E71.31, E71.32, E71.39, E75.21, E75.22, E75.23, E75.24, E75.25, E75.29, E75.3, E75.4, E75.5, E75.6, E77, E78.0, E78.1, E78.2, E78.3, E78.4, E78.5, E78.6, E78.70, E78.71, E78.72, E78.79, E78.8, E78.9
Coronary artery disease	398.91, 402, 404, 410-414, ICD-9-CM Procedure code (ICD-9-PCS): 36	I09.81, I11, I13, I20-I22, I24, I25.1-I25.7, I25.81, I25.89, ICD-10 Procedure code (ICD-10-PCS): 02
Stroke	430-438	G45.0, G45.1, G45.2, G45.3, G45.4, G45.8, G45.9, G46, I60, I61, I62, I63, I65, I66, I67.0, I67.1, I67.2, I67.3, I67.4, I67.5, I67.6, I67.7, I67.8, I67.9, I68, I69
Heart failure	428	I50
Atrial fibrillation	427	I45.0, I45.1, I45.2, I45.3, I45.4, I45.5, I45.6
Peripheral artery disease	440.0, 440.20, 440.21, 440.22, 440.23, 440.24, 440.3, 440.4, 443.9, 443.81, 443.89	I70.2, I70.92, I75.0, I73.9
Chronic obstructive pulmonary disease	491, 492, or 496	J41, J42, J44, J43, or J44.9
Alcohol-related disorders	291, 303, 305.0, 571.0-571.3, V11.3, V79.1	F10, K70.40, K70.41, K70.9
Liver cirrhosis	571.5, 571.2, 571.6	K70.2, K70.30, K70.31, K74.0, K74.1, K74.2, K74.60, K74.69, K74.3, K74.4, K74.5
Connective tissue diseases	710.0, 714.0, 725.0	M32.10, M06.9, M35.3
Family history of neoplasms	V16	Z80
Benign neoplasms	210-229	D10-D36, D3A
Psychosis	290-299	F20-29
Major depressive disorder	311	F32, F33
Dementia	290,290.4, 291.2, 292.82, 331	F03.90, F05, F02.80, F02.81, F01.50, F01.51, G30

Table S2 Characteristics for T2D patients without and with microvascular disease

Variables	No microvascular disease (N=387632)		Microvascular disease (N=387632)		SMD
	n	%	n	%	
Index year					
2009	185303	47.80	189320	48.84	0.021
2010	29117	7.51	28595	7.38	0.005
2011	22638	5.84	22208	5.73	0.005
2012	21240	5.48	20887	5.39	0.004
2013	20268	5.23	19784	5.10	0.006
2014	19686	5.08	19324	4.99	0.004
2015	17317	4.47	16858	4.35	0.006
2016	21107	5.45	20608	5.32	0.006
2017	24687	6.37	24526	6.33	0.002
2018	26269	6.78	25522	6.58	0.008
Sex					0.007
Female	182334	47.04	180967	46.69	
Male	205298	52.96	206665	53.31	
Age					
18-40	26042	6.72	27716	7.15	0.017
41-60	185812	47.94	185530	47.86	0.001
61-80	175778	45.35	174386	44.99	0.007
mean ± SD †	58.91	11.70	58.74	11.81	0.014
Comorbidities					
Overweight/Obesity	6493	1.68	6786	1.75	0.006
Smoking status	6557	1.69	6784	1.75	0.005
Hypertension	252983	65.26	251174	64.80	0.010
Dyslipidemia	231193	59.64	228709	59.00	0.013
Coronary artery disease	75001	19.35	74897	19.32	0.001
Stroke	21030	5.43	21455	5.53	0.005
Heart failure	17668	4.56	18692	4.82	0.012
Atrial fibrillation	26730	6.90	27457	7.08	0.007
Peripheral arterial disease	10626	2.74	11015	2.84	0.006
COPD	32838	8.47	33316	8.59	0.004
Alcohol related disorders	8145	2.10	8506	2.19	0.006
Liver cirrhosis	6800	1.75	6984	1.80	0.004
Connective tissue diseases	4831	1.25	5057	1.30	0.005
Family history of neoplasm	156	0.04	158	0.04	<0.001
Benign neoplasms	46717	12.05	47186	12.17	0.004
Psychosis	4994	1.29	5460	1.41	0.010
Major depressive disorder	11115	2.87	11739	3.03	0.010

Dementia	6875	1.77	7156	1.85	0.005
Medication					
Oral antidiabetic drugs					
Metformin	284770	73.46	278195	71.77	0.038
Sulfonylurea	239042	61.67	235004	60.63	0.021
Glinide	3288	0.85	3439	0.89	0.004
Thiazolidinedione	50639	13.06	50174	12.94	0.004
DPP-4 inhibitors	41279	10.65	40883	10.55	0.003
AGI	53390	13.77	53840	13.89	0.003
SGLT2 inhibitor	2650	0.68	2701	0.70	0.002
Numbers of oral antidiabetic drugs					
0-1	147838	38.14	153195	39.52	0.028
2-3	219567	56.64	214326	55.29	0.027
4-7	20227	5.22	20111	5.19	0.001
GLP-1 RA	379	0.10	411	0.11	0.003
Insulins	85798	22.13	88020	22.71	0.014
Antihypertensive drugs					
ACE inhibitors	105821	27.30	104762	27.03	0.006
ARBs	163131	42.08	162322	41.88	0.004
α-blockers	29714	7.67	30459	7.86	0.007
β-blockers	160921	41.51	160448	41.39	0.002
Calcium-channel blockers	204519	52.76	203712	52.55	0.004
Diuretics	129205	33.33	130310	33.62	0.006
Numbers of antihypertensive drugs					
0-1	163113	42.08	163240	42.11	0.001
2-3	142786	36.84	142241	36.69	0.003
4-6	81733	21.09	82151	21.19	0.003
Statin	177392	45.76	176057	45.42	0.007
Aspirin	152244	39.28	150764	38.89	0.008

Data are shown as n (%) or mean ± SD; SD: standard deviation.

Abbreviations: T2D, type 2 diabetes; SMD, standardized mean difference; COPD, chronic obstructive pulmonary disease; DPP-4, Dipeptidyl peptidase-4; AGI, Alpha-glucosidase inhibitors; SGLT2, sodium-glucose cotransporter 2; GLP-1RA, glucagon-like peptide-1 receptor agonist; ACE, angiotensin converting enzyme inhibitors; ARB, angiotensin receptor blockers., standardized A SMD < 0.1 indicates a negligible difference between the two groups of patients.

Table S3 Characteristics for T2D patients without and with one microvascular disease

Variables	No microvascular disease (N=340269)		One microvascular disease (N=340269)		SMD
	n	%	n	%	
Index year					
2009	159343	46.83	159640	46.92	0.002
2010	26500	7.79	25970	7.63	0.006
2011	20740	6.10	20226	5.94	0.006
2012	19242	5.65	18874	5.55	0.005
2013	18373	5.40	18039	5.30	0.004
2014	17867	5.25	17752	5.22	0.002
2015	15683	4.61	15409	4.53	0.004
2016	18704	5.50	18735	5.51	<0.001
2017	21376	6.28	22268	6.54	0.011
2018	22441	6.60	23356	6.86	0.011
Sex					0.007
Female	160006	47.02	158755	46.66	
Male	180263	52.98	181514	53.34	
Age					
18-40	23683	6.96	25316	7.44	0.019
41-60	164741	48.41	163535	48.06	0.007
61-80	151845	44.62	151418	44.50	0.003
mean ± SD †	58.69	11.73	58.58	11.90	0.010
Comorbidities					
Overweight/Obesity	5730	1.68	6240	1.83	0.011
Smoking status	5612	1.65	6112	1.80	0.011
Hypertension	219342	64.46	217331	63.87	0.012
Dyslipidemia	202832	59.61	201775	59.30	0.006
Coronary artery disease	64084	18.83	64468	18.95	0.003
Stroke	17084	5.02	17796	5.23	0.009
Heart failure	14481	4.26	15131	4.45	0.009
Atrial fibrillation	22962	6.75	24322	7.15	0.016
Peripheral arterial disease	8701	2.56	9154	2.69	0.008
COPD	28120	8.26	29349	8.63	0.013
Alcohol related disorders	6973	2.05	7567	2.22	0.012
Liver cirrhosis	5764	1.69	5981	1.76	0.005
Connective tissue diseases	4099	1.20	4495	1.32	0.010
Family history of neoplasm	138	0.04	150	0.04	0.002
Benign neoplasms	41624	12.23	42971	12.63	0.012
Psychosis	4407	1.30	4932	1.45	0.013
Major depressive disorder	9466	2.78	10328	3.04	0.015

Dementia	5720	1.68	6174	1.81	0.010
Medication					
Oral antidiabetic drugs					
Metformin	245680	72.20	241875	71.08	0.025
Sulfonylurea	204389	60.07	200956	59.06	0.021
Glinide	2636	0.77	2843	0.84	0.007
Thiazolidinedione	41419	12.17	40699	11.96	0.006
DPP-4 inhibitors	33972	9.98	34802	10.23	0.008
AGI	43722	12.85	43717	12.85	<0.001
SGLT2 inhibitor	2050	0.60	2418	0.71	0.013
Numbers of oral antidiabetic drugs					
0-1	136442	40.10	140013	41.15	0.021
2-3	187904	55.22	184265	54.15	0.021
4-7	15923	4.68	15991	4.70	0.001
GLP-1 RA	274	0.08	340	0.10	0.006
Insulins	68250	20.06	68470	20.12	0.002
Antihypertensive drugs					
ACE inhibitors	89859	26.41	88544	26.02	0.009
ARBs	139098	40.88	137783	40.49	0.008
α-blockers	24799	7.29	25408	7.47	0.007
β-blockers	139470	40.99	139308	40.94	0.001
Calcium-channel blockers	176877	51.98	175559	51.59	0.008
Diuretics	109706	32.24	109745	32.25	<0.001
Numbers of antihypertensive drugs					
0-1	147172	43.25	147843	43.45	0.004
2-3	124355	36.55	124029	36.45	0.002
4-6	68742	20.20	68397	20.10	0.003
Statin	153361	45.07	152405	44.79	0.006
Aspirin	129975	38.20	129372	38.02	0.004

† Data are shown as n (%) or mean ± SD; SD: standard deviation.

Abbreviations: T2D, type 2 diabetes; SMD, standardized mean difference; COPD, chronic obstructive pulmonary disease; DPP-4, Dipeptidyl peptidase-4; AGI, Alpha-glucosidase inhibitors; SGLT2, sodium-glucose cotransporter 2; GLP-1RA, glucagon-like peptide-1 receptor agonist; ACE, angiotensin converting enzyme inhibitors; ARB, angiotensin receptor blockers., standardized A SMD < 0.1 indicates a negligible difference between the two groups of patients.

Table S4 Characteristics for T2D patients without and with two microvascular diseases

Variables	No microvascular disease (N=43109)		Two microvascular diseases (N=43109)		SMD
	n	%	n	%	
Index year					
2009	23585	54.71	26631	61.78	0.144
2010	2398	5.56	2407	5.58	0.001
2011	1773	4.11	1864	4.32	0.011
2012	1865	4.33	1868	4.33	<0.001
2013	1728	4.01	1615	3.75	0.014
2014	1678	3.89	1447	3.36	0.029
2015	1518	3.52	1344	3.12	0.023
2016	2179	5.05	1764	4.09	0.046
2017	2969	6.89	2135	4.95	0.082
2018	3416	7.92	2034	4.72	0.132
Sex					0.005
Female	20377	47.27	20280	47.04	
Male	22732	52.73	22829	52.96	
Age					
18-40	2170	5.03	2214	5.14	0.005
41-60	19212	44.57	19789	45.90	0.027
61-80	21727	50.40	21106	48.96	0.029
mean ± SD †	60.38	11.37	60.01	11.21	0.032
Comorbidities					
Overweight/Obesity	693	1.61	519	1.20	0.034
Smoking status	858	1.99	620	1.44	0.043
Hypertension	30425	70.58	30579	70.93	0.008
Dyslipidemia	25780	59.80	24580	57.02	0.057
Coronary artery disease	9769	22.66	9380	21.76	0.022
Stroke	3491	8.10	3240	7.52	0.022
Heart failure	2806	6.51	3099	7.19	0.027
Atrial fibrillation	3376	7.83	2898	6.72	0.043
Peripheral arterial disease	1705	3.96	1655	3.84	0.006
COPD	4223	9.80	3673	8.52	0.044
Alcohol related disorders	1055	2.45	868	2.01	0.029
Liver cirrhosis	917	2.13	887	2.06	0.005
Connective tissue diseases	654	1.52	517	1.20	0.027
Family history of neoplasm					0.012
Benign neoplasms	4660	10.81	3951	9.17	0.055
Psychosis	528	1.22	488	1.13	0.009
Major depressive disorder	1461	3.39	1290	2.99	0.023

Dementia	1046	2.43	920	2.13	0.020
Medication					
Oral antidiabetic drugs					
Metformin	35424	82.17	33234	77.09	0.126
Sulfonylurea	31310	72.63	30917	71.72	0.020
Glinide	574	1.33	536	1.24	0.008
Thiazolidinedione	8179	18.97	8450	19.60	0.016
DPP-4 inhibitors	6415	14.88	5485	12.72	0.063
AGI	8560	19.86	8924	20.70	0.021
SGLT2 inhibitor	533	1.24	269	0.62	0.064
Numbers of oral antidiabetic drugs					
0-1	10711	24.85	12040	27.93	0.070
2-3	28645	66.45	27467	63.72	0.057
4-7	3753	8.71	3602	8.36	0.013
GLP-1 RA	93	0.22	65	0.15	0.015
Insulins	15281	35.45	16822	39.02	0.074
Antihypertensive drugs					
ACE inhibitors	14307	33.19	14474	33.58	0.008
ARBs	21555	50.00	21875	50.74	0.015
α-blockers	4346	10.08	4408	10.23	0.005
β-blockers	19348	44.88	19012	44.10	0.016
Calcium-channel blockers	24970	57.92	25275	58.63	0.014
Diuretics	17420	40.41	18182	42.18	0.036
Numbers of antihypertensive drugs					
0-1	14772	34.27	14437	33.49	0.016
2-3	16809	38.99	16626	38.57	0.009
4-6	11528	26.74	12046	27.94	0.027
Statin	21685	50.30	21283	49.37	0.019
Aspirin	20020	46.44	19240	44.63	0.036

† Data are shown as n (%) or mean ± SD; SD: standard deviation.

Abbreviations: T2D, type 2 diabetes; SMD, standardized mean difference; COPD, chronic obstructive pulmonary disease; DPP-4, Dipeptidyl peptidase-4; AGI, Alpha-glucosidase inhibitors; SGLT2, sodium-glucose cotransporter 2; GLP-1RA, glucagon-like peptide-1 receptor agonist; ACE, angiotensin converting enzyme inhibitors; ARB, angiotensin receptor blockers., standardized A SMD < 0.1 indicates a negligible difference between the two groups of patients.

Table S5 Characteristics for T2D patients without and with three microvascular diseases

Variables	No microvascular disease (N=4254)		Three microvascular diseases (N=4254)		SMD
	n	%	n	%	
Index year					
2009	2375	55.83	3049	71.67	0.334
2010	219	5.15	218	5.12	0.001
2011	125	2.94	118	2.77	0.010
2012	133	3.13	145	3.41	0.016
2013	167	3.93	130	3.06	0.047
2014	141	3.31	125	2.94	0.022
2015	116	2.73	105	2.47	0.016
2016	224	5.27	109	2.56	0.140
2017	342	8.04	123	2.89	0.228
2018	412	9.69	132	3.10	0.272
Sex					0.009
Female	1951	45.86	1932	45.42	
Male	2303	54.14	2322	54.58	
Age					
18-40	189	4.44	186	4.37	0.003
41-60	1859	43.70	2206	51.86	0.164
61-80	2206	51.86	1862	43.77	0.162
mean ± SD †	60.97	11.24	58.90	10.41	0.191
Comorbidities					
Overweight/Obesity	70	1.65	27	0.63	0.095
Smoking status	87	2.05	52	1.22	0.065
Hypertension	3216	75.60	3264	76.73	0.026
Dyslipidemia	2581	60.67	2354	55.34	0.108
Coronary artery disease	1148	26.99	1049	24.66	0.053
Stroke	455	10.70	419	9.85	0.028
Heart failure	381	8.96	462	10.86	0.064
Atrial fibrillation	392	9.21	237	5.57	0.140
Peripheral arterial disease	220	5.17	206	4.84	0.015
COPD	495	11.64	294	6.91	0.163
Alcohol related disorders	117	2.75	71	1.67	0.074
Liver cirrhosis	119	2.80	116	2.73	0.004
Connective tissue diseases	78	1.83	45	1.06	0.065
Family history of neoplasm					0.022
Benign neoplasms	433	10.18	264	6.21	0.145
Psychosis	59	1.39	40	0.94	0.042
Major depressive disorder	188	4.42	121	2.84	0.084

Dementia	109	2.56	62	1.46	0.079
Medication					
Oral antidiabetic drugs					
Metformin	3666	86.18	3086	72.54	0.342
Sulfonylurea	3343	78.58	3131	73.60	0.117
Glinide	78	1.83	60	1.41	0.034
Thiazolidinedione	1041	24.47	1025	24.09	0.009
DPP-4 inhibitors	892	20.97	596	14.01	0.184
AGI	1108	26.05	1199	28.19	0.048
SGLT2 inhibitor	67	1.57	14	0.33	0.129
Numbers of oral antidiabetic drugs					
0-1	685	16.10	1142	26.85	0.264
2-3	3018	70.94	2594	60.98	0.212
4-7	551	12.95	518	12.18	0.023
GLP-1 RA	12	0.28	6	0.14	0.031
Insulins	2267	53.29	2728	64.13	0.221
Antihypertensive drugs					
ACE inhibitors	1655	38.90	1744	41.00	0.043
ARBs	2478	58.25	2664	62.62	0.090
α-blockers	569	13.38	643	15.12	0.050
β-blockers	2103	49.44	2128	50.02	0.012
Calcium-channel blockers	2672	62.81	2878	67.65	0.102
Diuretics	2079	48.87	2383	56.02	0.143
Numbers of antihypertensive drugs					
0-1	1169	27.48	960	22.57	0.114
2-3	1622	38.13	1586	37.28	0.017
4-6	1463	34.39	1708	40.15	0.119
Statin	2346	55.15	2369	55.69	0.011
Aspirin	2249	52.87	2152	50.59	0.046

†Data are shown as n (%) or mean ± SD; SD: standard deviation.

Abbreviations: T2D, type 2 diabetes; SMD, standardized mean difference; COPD, chronic obstructive pulmonary disease; DPP-4, Dipeptidyl peptidase-4; AGI, Alpha-glucosidase inhibitors; SGLT2, sodium-glucose cotransporter 2; GLP-1RA, glucagon-like peptide-1 receptor agonist; ACE, angiotensin converting enzyme inhibitors; ARB, angiotensin receptor blockers., standardized A SMD < 0.1 indicates a negligible difference between the two groups of patients.
